# Anaesthetic Management of Advanced Late-Onset Pompe Disease: Challenges in a Major Abdominal Surgery

**DOI:** 10.7759/cureus.93349

**Published:** 2025-09-27

**Authors:** Maria Silva, Maria Fátima Santos, Germano Cardoso

**Affiliations:** 1 Department of Anesthesiology, Unidade Local de Saúde de Santo António, Porto, PRT; 2 Department of Anesthesiology, Instituto Português de Oncologia do Porto Francisco Gentil, Porto, PRT

**Keywords:** glycogen storage disease type ii, late-onset pompe disease, major open abdominal surgery, perioperative respiratory management, regional analgesia

## Abstract

Pompe disease is a rare autosomal recessive lysosomal storage disorder caused by a deficiency of acid alpha-glucosidase, leading to glycogen accumulation in various tissues. The late-onset form predominantly affects skeletal and respiratory muscles, with minimal cardiac involvement. Due to respiratory muscle weakness and potential pulmonary complications, this disease presents significant challenges in perioperative management. We report the case of a patient with late-onset Pompe disease with moderate restrictive disease, dependent on nocturnal bilevel positive airway pressure (BiPAP), who underwent elective major abdominal surgery under combined general and epidural anaesthesia. This case emphasises the importance of multidisciplinary perioperative management, which includes postoperative respiratory support strategies and the choice of regional analgesia. Despite the rarity of such cases, especially in major non-cardiac surgery, this report contributes valuable insights into safe anaesthetic approaches for this complex patient population.

## Introduction

Pompe disease, or glycogen storage disease type II, is a rare autosomal recessive disorder caused by the deficiency of the lysosomal enzyme acid alpha-glucosidase, which is responsible for the degradation of glycogen polymers. The secondary lysosomal and cytoplasmic accumulation of glycogen occurs predominantly in skeletal, cardiac, and smooth muscle [1].

This disease has been described in the literature based on age of onset and severity, classified as infantile-onset Pompe disease (IOPD) and late-onset Pompe disease (LOPD). IOPD typically presents with severe cardiac involvement, including hypertrophic cardiomyopathy, hypotonia, and respiratory insufficiency, and is often fatal within the first year of life without treatment. In contrast, LOPD, which is the focus of this report, usually has a more favorable short-term prognosis. It may present at any age, most often after the first year, and follows an insidious course, manifesting as progressive muscle weakness, characterized by the absence of severe cardiac involvement. [2] Life expectancy depends on disease progression, degree of respiratory muscle involvement, and associated comorbidities [1,2].

The pathophysiological features of LOPD translate directly into perioperative challenges. Patients may require long-term ventilatory support and are at increased risk of perioperative complications due to diaphragmatic and intercostal muscle weakness and impaired airway clearance. Sleep-disordered breathing and intolerance to the supine position may complicate induction and extubation, while bulbar involvement can increase aspiration risk. Sensitivity to neuromuscular blocking agents and the potential for prolonged weakness are additional concerns [2,3].

Because of these multisystem implications, anaesthetic management is complex, with limited literature to guide practice in major abdominal surgery.

We present a case of a 55-year-old man with LOPD, dependent on nocturnal bilevel positive airway pressure (BiPAP), undergoing elective open abdominal surgery under combined general and epidural anaesthesia. This case highlights key anaesthetic considerations in the management of this disease, including the role of respiratory support strategies, regional analgesia, and postoperative planning.

## Case presentation

A 55-year-old man with a history of Pompe disease was scheduled for major abdominal surgery.

**Figure 1:**
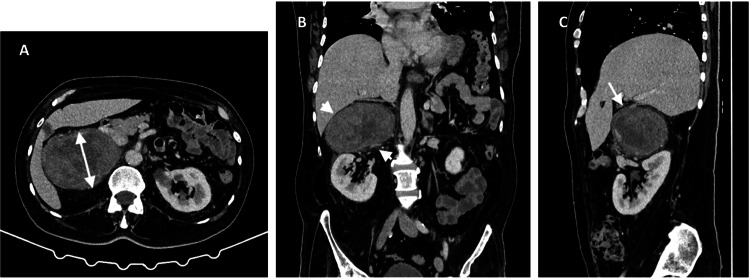
Infrahepatic mass in the region of the adrenal gland visible on the CT scan. The image shows an infrahepatic mass measuring 11.5 × 8.7 × 7.9 cm, indicated by arrows in a CT scan in axial (A), coronal (B), and sagittal (C) planes.

The diagnosis of Pompe disease was established in 2014, and the patient had been followed by pneumology and neuromuscular specialist teams. Initially, he presented with muscle weakness and proximal tetraparesis, which progressed to difficulties with ambulation and standing without assistance, leading to early retirement due to disability. He had been using BiPAP support for over five years and was unable to tolerate the supine position without it. No bulbar symptoms or gastroesophageal reflux were reported. He had been receiving enzyme replacement therapy with alglucosidase alfa every two weeks since the diagnosis. Due to abdominal pain, an abdominal CT scan was performed, revealing an infrahepatic mass near the adrenal gland, measuring 11.5 × 8.7 × 7.9 cm (Figure 1), suspicious for either a paraganglioma or sarcoma. Functional studies, including plasma and urinary metanephrines, were performed to rule out a functional pheochromocytoma given the adrenal mass location or a functional paraganglioma, and the results were normal. The patient was scheduled for resection of the mass located in the adrenal fossa via laparotomy.

**Figure 2:**
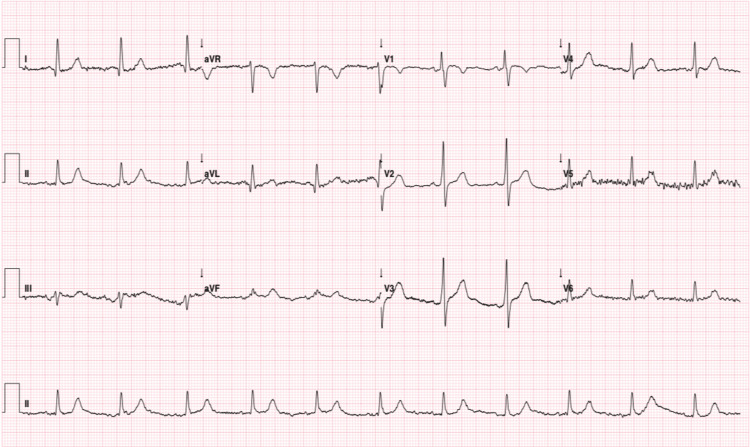
Preoperative electrocardiogram. Electrocardiogram showing sinus rhythm at 67 bpm with a mild nonspecific intraventricular conduction delay.

**Figure 3:**
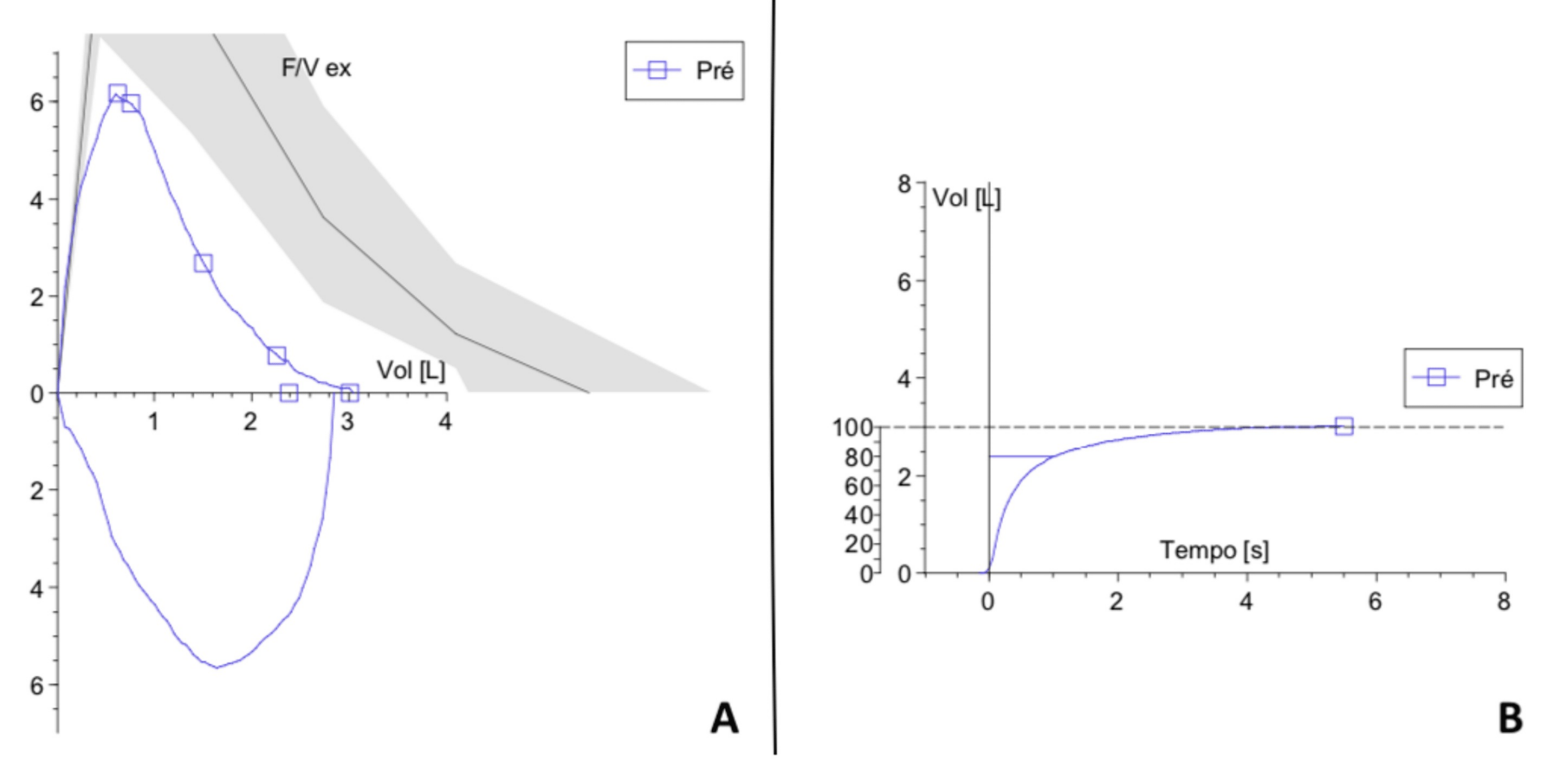
Flow-volume loop (A) and volume-time curve (B). Graph A shows the flow-volume loop with a restrictive pattern (reduced FVC and FEV₁, preserved ratio FEV1/FVC). Graph B shows the volume-time curve with reduced FVC and normal plateau.

As part of the preoperative workup, transthoracic echocardiography revealed moderate aortic regurgitation (vena contracta width of 3.3 mm), with preserved left ventricular systolic function (ejection fraction of 66%), and no evidence of hypertrophy or obstructive cardiomyopathy. The electrocardiogram showed no significant abnormalities (Figure 2). Spirometry demonstrated a moderate restrictive ventilatory pattern, according to ERS/ATS 2022 guidelines [4], with a forced vital capacity (FVC) of 55% and forced expiratory volume in one second (FEV₁) of 56% (Figure 3 and Table 1). Polysomnography could not be completed without BiPAP support; the most recent study demonstrated an apnoea-hypopnoea index (AHI) of 17.9 per hour, with oxygen saturation levels below 90% for a total of 88 minutes.

**Table 1:** Spirometry results. FVC: Forced Vital Capacity; FEV₁: Forced Expiratory Volume in 1 second; FEV₁/FVC: ratio of FEV₁ to FVC; PEF: Peak Expiratory Flow; % Predicted and Z-score values are calculated based on age, sex, height, and ethnicity reference equations. Interpretation of results follows the ERS/ATS 2022 criteria [4].

Variables	Measured Value	% Predicted	Z-Score	Interpretation
FVC (L)	3.0	55%	-3.2	Reduced
FEV1 (L)	2.38	56%	-3.0	Reduced
FEV1/FVC (%)	79.1	-	0.2	Normal
PEF (L/s)	6.1	66%	-2.6	Reduced

During the pre-anesthetic assessment, it was noted that he required BiPAP when in the supine position (even while awake) and occasionally during the day. On examination, he weighed 88 kg and measured 1.87 m in height. Airway evaluation revealed a Mallampati class II, with adequate mouth opening and neck mobility. Cardiopulmonary auscultation was clear with no additional sounds. Oxygen saturation was 97% on room air. Laboratory tests, including hemoglobin, renal function, and electrolytes, showed no abnormalities. A combined general and epidural anesthesia was proposed, and informed consent was obtained.

In the operating room, the patient was monitored according to American Society of Anesthesiologists (ASA) standards, including esophageal temperature monitoring. Additional monitoring included invasive arterial blood pressure, urine output, processed electroencephalography (pEEG) using SedLine® Brain Function Monitor (Masimo Corporation, Irvine, CA), and neuromuscular blockade assessment with train-of-four (TOF) stimulation.

Before induction, the epidural catheter was successfully placed on the first attempt at the thoracic level (T9-T10) in the lateral decubitus position with loss of resistance to saline.

General anaesthesia was induced after adequate pre-oxygenation with continuous positive airway pressure (CPAP), by the slow administration of 100 mcg of fentanyl, 100 mg of propofol, and 40 mg of rocuronium. Orotracheal intubation was performed via direct laryngoscopy without complications. The patient remained haemodynamically stable throughout the induction.

A central venous catheter was then placed in the right internal jugular vein using an ultrasound-guided technique. Prior to the surgical incision, 10 µg of sufentanyl and 6 mL of 0.1% ropivacaine were administered via the epidural catheter.

Anaesthesia was maintained with sevoflurane titrated to maintain Patient State Index (PSI) values between 25 and 50, and nociception was controlled with intermittent epidural boluses of 6 mL of 0.1% ropivacaine. Neuromuscular relaxants were administered as needed based on surgical requirements and TOF monitoring. Body temperature was maintained around 36°C by active forced hot air blankets. Ventilation was volume-controlled, with a tidal volume of 480 mL, respiratory rate of 16 breaths per minute, and PEEP of 7 cmH₂O, maintaining an EtCO2 between 35 and 39 mmHg.

The procedure lasted approximately 180 minutes without complications. The patient remained hemodynamically stable, and final arterial blood gas values were as follows: pH 7.38 (7.35-7.45), PaCO₂ 40 mmHg (35-45 mmHg), PaO₂ 167 mmHg (80-100 mmHg), lactate 0.6 mmol/L (0.5-2.0 mmol/L), HCO₃⁻ 23.7 mmol/L (22-26 mmol/L), and hemoglobin 13.3 g/dL (Table 2). Estimated blood loss was 450 mL. Approximately 2 L of balanced crystalloid solution was infused.

**Table 2:** Arterial blood gas analysis.

Arterial Blood Gas	Intraoperative (invasive ventilation)	Postoperative (spontaneous ventilation)	Reference
FiO_2_	0,40	0.21	-
pH	7.38	7.45	7.35-7.45
PaO_2_	167	76	80-100 mmHg
PaCO_2_	40	34	35-45 mmHg
HCO_3_^-^	23.7	25	22-26 mmol/L
Lactate	0.6	1.0	0.5-2.0 mmol/L

At the end of the procedure, the TOF count at the time of reversal was 1/4. Sugammadex was administered at a dose of 2 mg/kg, resulting in a TOF ratio >0.9. Sevoflurane was then discontinued, and the patient was extubated directly onto his personal BiPAP device and transferred to the intensive care unit (ICU) for continued invasive monitoring.

During the first postoperative day and night, he remained on his usual BiPAP settings (iPAP 18 cmH₂O and ePAP 8 cmH₂O), with no incidents occurring. Analgesia was maintained with a continuous epidural infusion of 0.1% ropivacaine with 0.001% sufentanil at 6ml/h, supplemented by patient-controlled 5ml boluses during the first three postoperative days. Systemic analgesia consisted of paracetamol every six hours.

On the second postoperative day, daytime BiPAP support was discontinued, and the patient maintained spontaneous ventilation without supplemental oxygen. Blood gas showed pH 7.45 (7.35-7.45), PaO2 76 mmHg (80-100mmHg), PaCO2 34 mmHg (35-45 mmHg), HCO3- 25 mmEq (22-26 mmol/L). The results of blood gas analyses during the intraoperative and postoperative period were summarized in Table 2.

At the end of the second postoperative day, he was discharged from the ICU to the surgical ward, where he remained for six days before being discharged home without complications.

Histopathological analysis revealed findings consistent with a cellular schwannoma. At the 30-day follow-up consultation after hospital discharge, the patient reported no complications and had resumed his pre-admission activities without additional limitations.

## Discussion

​​​​​​Pompe disease, particularly its late-onset form, presents significant anaesthetic challenges due to its multisystem involvement, most notably affecting the respiratory system, and the lack of universally established standards or guidelines for perioperative management.

LOPD is characterised by a restrictive pattern on spirometry, an unproductive coughing with frequent infections, blood gas disturbances, and sleep-disordered breathing, with an associated risk of aspiration pneumonia and respiratory failure [2].

Proximal lower limb and paraspinal trunk muscles are usually affected first, followed by involvement of the diaphragm and accessory muscles of respiration. As the muscle weakness worsens, patients often become wheelchair users and may require assisted ventilation. Respiratory failure, usually a consequence of respiratory tract infection, is a major cause of morbidity and mortality in this form of the disease [2,3,5].

In patients with Pompe disease, pulmonary function is often more compromised in the supine position than in the upright position due to severe diaphragmatic weakness [1,5]. Approximately 30-40% of patients exhibit a moderate reduction in vital capacity, as in our case, while the remaining 60% typically show only mild impairment. Since the decline in respiratory function can precede the decline in locomotor function, it is important to perform annual evaluations of pulmonary function [2,5]. This patient presented with a moderate reduction in vital capacity, along with advanced diaphragmatic and intercostal muscle weakness, requiring BiPAP support when lying down.

Sleep-disordered breathing is frequently observed due to the mechanical disadvantage of the supine position and the natural reduction in respiratory drive during sleep. A decrease in upper airway tone, particularly during rapid eye movement (REM) sleep, may further compromise ventilation. Nocturnal hypoventilation commonly precedes daytime respiratory failure and may occur even when upright vital capacity is only moderately reduced, reflecting disproportionate diaphragmatic involvement. Polysomnography should be performed at baseline and then again when the FVC declines [2,5]. In this case, the patient was unable to tolerate preoperative polysomnography without non-invasive ventilation, supporting the presence of this comorbidity. Although progression to cor pulmonale and cardiorespiratory failure is possible in advanced cases, no such findings were evident in our patient [2].

Because a major concern in these patients is the increased risk of postoperative respiratory failure and pulmonary complications, regional anaesthesia is preferred over general anaesthesia and should be considered the technique of choice. No complications directly related to regional techniques have been reported [6-8]. In this case, it was not possible to avoid general anaesthesia; however, the anaesthetic plan was designed to minimise respiratory depression through the use of combined general and epidural anaesthesia. The epidural technique reduced the need for systemic opioids and provided effective analgesia, facilitating faster emergence, earlier extubation to BiPAP, and preserving cough effectiveness for secretion clearance. Postoperative pain control with a low-concentration local anaesthetic epidural infusion facilitated effective respiration and chest physiotherapy, thereby reducing the risk of atelectasis [7,9].

In patients with Pompe disease, a thorough preoperative evaluation should include transthoracic echocardiography, even though hypertrophic cardiomyopathy and conduction disturbances are less commonly associated with the late-onset form [1-3]. In our patient, moderate aortic regurgitation was identified, but systolic function was preserved without ventricular hypertrophy. Therefore, haemodynamic instability following induction was not a major concern in this case, in contrast to other reports where cardiac involvement, such as hypertrophic cardiomyopathy, posed greater risk [6,7]. Induction was performed using fentanyl and propofol administered over an extended period, without any resulting cardiovascular instability.

Because of the underlying muscle weakness, Pompe patients may be more sensitive to neuromuscular blockade. Agents such as suxamethonium should be avoided in Pompe disease, as in all myopathic patients, due to the potential risk of rhabdomyolysis and hyperkalaemia [2,7,10]. In this case, neuromuscular blockade was achieved with rocuronium, with quantitative monitoring by TOF, and reversal with sugammadex enabled safe extubation. This highlights the value of quantitative neuromuscular monitoring and the availability of specific reversal agents.

Feeding and swallowing difficulties, often leading to challenges in maintaining normal weight, are common symptoms in Pompe disease [2]. These may be of particular concern for the anaesthesiologist if gastroesophageal reflux is present, due to the increased risk of aspiration. In this patient, however, this concern was neither documented nor reported by the patient.

When patients with Pompe disease are hospitalised, the preferred setting is a respiratory intensive care unit. Management typically includes standard measures, such as non-invasive positive pressure ventilation to support inspiratory muscles, with endotracheal intubation reserved for severe cases; maintaining patients in a semi-upright position, as supine posture may worsen respiratory mechanics; oxygen supplementation to maintain saturations between 92% and 94%, along with close monitoring of PaCO₂ and pH; airway clearance using cough-assist devices and physiotherapy techniques; nutritional support to reduce aspiration risk; and prompt treatment of respiratory infections [3,5]. In this case, it was essential to develop a meticulous postoperative plan, including the availability of an ICU support and preparation for the possible need for invasive ventilation in the immediate postoperative period. However, the favourable outcome of this case depended on early extubation.

Table 3 summarises the key points to consider in the perioperative care of patients with LOPD, particularly addressing risks related to respiratory, cardiac, and neuromuscular functions.

**Table 3:** Key perioperative considerations for patients with late-onset Pompe disease. Adapted from [2,4,5] BiPAP: bilevel positive airway pressure

Perioperative Considerations for Patients with Late-Onset Pompe Disease		
Preoperative	General Clinical Evaluation	Review of disease history: onset, progression, respiratory and muscular symptoms and home medication.
		Presence of muscle weakness and respiratory symptoms
		Intolerance to supine position
		Aspiration risk by bulbar symptoms or gastroesophageal reflux
		Nutritional status (BMI, weight trends)
		Functional status and level of dependence
	Respiratory Assessment	Pulmonary function tests (FVC, FEV₁)
		Baseline arterial blood gas
		Polysomnography (if available)
		- BiPAP usage (nocturnal/daytime) and weaning tolerance
		Pulmonology consultation recommended
	Cardiac Assessment	Echocardiography (ejection fraction and cardiomyopathy)
		ECG (arrhythmias, conduction disturbances)
		Cardiology consultation if indicated
Intraoperative	Anaesthetic Planning	Multidisciplinary discussion (anaesthesiology, surgery, and intensive care)
		Avoid general anesthesia. Consider regional anaesthesia if possible.
		Opioid-sparing analgesia strategy
		Neuromuscular monitoring and titrated doses of muscle relaxants.
		Reversal agent plan (prefer sugammadex, avoid succinylcholine)
Postoperative	Postoperative Planning	ICU or monitored setting available
		BiPAP device ready and functional
		Pulmonary physiotherapy and secretion clearance strategy

## Conclusions

We consider this case report important, as anaesthetic management in patients with Pompe disease is rare, and the available evidence is limited, mostly restricted to individual case reports. Regional anesthesia has been used successfully in several reports without complications and is often considered the preferred approach. However, in the context of major abdominal surgery, as in this case, regional anaesthesia alone was not a feasible option, and general anaesthesia with invasive ventilation was required.

Therefore, anaesthetic management of patients with LOPD should prioritise respiratory support, opioid-sparing strategies, vigilant neuromuscular monitoring, and a multidisciplinary approach. Combined general and regional anaesthesia may offer advantages in major surgery, contributing to a favourable postoperative course.
